# Suppressed phase separation of mixed-halide perovskites confined in endotaxial matrices

**DOI:** 10.1038/s41467-019-08610-6

**Published:** 2019-02-11

**Authors:** Xi Wang, Yichuan Ling, Xiujun Lian, Yan Xin, Kamal B. Dhungana, Fernando Perez-Orive, Javon Knox, Zhizhong Chen, Yan Zhou, Drake Beery, Kenneth Hanson, Jian Shi, Shangchao Lin, Hanwei Gao

**Affiliations:** 1 Department of Physics, Florida State University, Tallahassee, FL 32306 USA; 2 Condensed Matter Science, National High Magnetic Field Laboratory, Tallahassee, FL 32310 USA; 3 Department of Mechanical Engineering, Florida State University, Tallahassee, FL 32310 USA; 4 Department of Materials Science and Engineering, Rensselaer Polytechnic Institute, Troy, NY 12180 USA; 5 Department of Chemistry and Biochemistry, Florida State University, Tallahassee, FL 32306 USA; 6 Materials Science & Engineering Program, Florida State University, Tallahassee, FL 32306 USA; 7 School of Mechanical and Power Engineering, Shanghai Jiao Tong University, 200240 Shanghai, China

## Abstract

The functionality and performance of a semiconductor is determined by its bandgap. Alloying, as for instance in In_x_Ga_1-x_N, has been a mainstream strategy for tuning the bandgap. Keeping the semiconductor alloys in the miscibility gap (being homogeneous), however, is non-trivial. This challenge is now being extended to halide perovskites – an emerging class of photovoltaic materials. While the bandgap can be conveniently tuned by mixing different halogen ions, as in CsPb(Br_x_I_1-x_)_3_, the so-called mixed-halide perovskites suffer from severe phase separation under illumination. Here, we discover that such phase separation can be highly suppressed by embedding nanocrystals of mixed-halide perovskites in an endotaxial matrix. The tuned bandgap remains remarkably stable under extremely intensive illumination. The agreement between the experiments and a nucleation model suggests that the size of the nanocrystals and the host-guest interfaces are critical for the photo-stability. The stabilized bandgap will be essential for the development of perovskite-based optoelectronics, such as tandem solar cells and full-color LEDs.

## Abstract (web summary)

The bandgap of mixed-halide perovskites can be continuously tuned by changing the halide ratio, but the crystals have poor photo-stability. Here the authors show that photoinduced phase separation is suppressed when perovskite nanocrystals are embedded in a non-perovskite endotaxial matrix.

## Introduction

The earth-abundant composition and the simple synthetic procedures make halide perovskites promising for the effective optoelectronics^1–8^, such as solution-processed high-efficiency solar cells^9–13^ and high-brightness light emitting diodes^14–18^. Besides other desirable optical and electrical properties, the halide perovskites offer a unique opportunity to optimize the spectral range of photoresponse by tuning the bandgaps^19,20^. Two types of halogen anions can be accommodated in homogenous crystal lattices with nearly arbitrary mixing ratios, leading to a wide range of the bandgap tunability. For example, the bandgap of the perovskite CsPb(Br_x_I_1-x_)_3_ can be tuned continuously from 2.4 eV (corresponding to CsPbBr_3_) to 1.75 eV (corresponding to CsPbI_3_), covering half of the visible spectrum. The range could be further extended when chlorine is incorporated^19,20^. In optoelectronic applications, the bandgap of photoactive semiconductors is often critical to device performance—it determines the upper limit of the energy conversion efficiency in solar cells^21^, or the color of emission in light emitting diodes^17,18^.

The practical value of the bandgap tunability in perovskites, however, has been limited due to poor material stability under optical illumination^22–28^. While the freshly prepared mixed-halide perovskites exhibit homogenous crystal phases, the bandgap could vary significantly under continuous optical illumination. Various measurements indicate that the observed bandgap shifts could be attributed to photoinduced phase separation^22–28^. The homogeneously distributed halogen anions segregated into bromine- and iodine-rich domains, exhibiting a redshifted photoluminescence peak and split X-ray diffraction patterns^22^. A few studies show that such photoinduced degradation could be mitigated in some of the mixed-halide perovskites with certain compositions^18,29^. Yet, stabilizing the bandgap across the entire spectrum (from pure bromide to pure iodide, i.e. 0 < *x* < 1) remains challenging. In this work, we find that the light-induced phase separation could be effectively suppressed when crystals of the mixed-halide perovskite CsPb(Br_x_I_1-x_)_3_ are spatially confined in an endotaxial matrix Cs_4_Pb(Br_x_I_1-x_)_6_. Stabilization of the tuned bandgap is achieved across the full spectrum (regardless of the Br/I ratio). It is remarkable that, for certain composition (*x* ≤ 0.6), the tuned bandgaps remain stable under extremely intensive illumination up to 440 W cm^−2^ (as a reference, the standard solar spectrum (AM1.5) has an integrated power density of 0.1 W cm^−2^). Note that such intensity is about an order of magnitude higher than what is typically used in concentrator solar cells^30^. The observed phenomena are consistent with the predictions from a thermodynamic model based on nucleation in solids. In both experiments and theory, the size of the crystallites and the host-guest cohesive energy, are found to be critical factors to the photo-stability of the mixed-halide perovskites.

## Results

### Correlation between the phase stability and the morphology

**Fig. 1:**
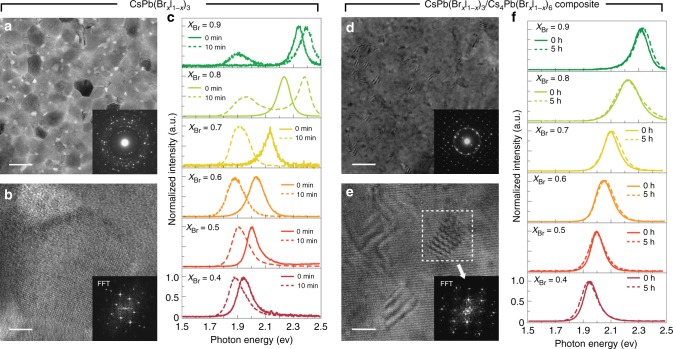
The phase stability of mixed-halide perovskite CsPb(Br_x_I_1-x_)_3_ is correlated with the morphology. **a** A representative TEM image of polycrystalline CsPb(Br_x_I_1-x_)_3_ thin films showed an average domain size around *r* = 35 nm. **b** An HR-TEM image of a single domain and the corresponding FFT pattern of the image (inset) confirmed these thin films were composed of perovskites. **c** The photoluminescence peak of these mixed-halide perovskites shifted exclusively to 1.87 eV after continuous illumination. The solid lines were the spectra taken from freshly made samples and the dashed lines were measured after 10 min illumination with the intensity of 0.3 W cm^−2^. **d** In composite thin films, CsPb(Br_x_I_1-x_)_3_ and Cs_4_Pb(Br_x_I_1-x_)_6_ formed host-guest structures. Inset: the electron diffraction pattern was dominated by a Cs_4_Pb(Br_x_I_1-x_)_6_ single crystal, where the ring came from the distributed CsPb(Br_x_I_1-x_)_3_ crystallites. **e** An HR-TEM image with clear Moiré Fringes showed an average CsPb(Br_x_I_1-x_)_3_ domain size about *r* = 7.5 nm. Inset: the FFT pattern of the highlight area confirmed that the Moiré Fringes were formed by overlapping the lattices of the Cs_4_Pb(Br_x_I_1-x_)_6_ host and the CsPb(Br_x_I_1-x_)_3_ guest. **f** The wavelength-tunable photoluminescence from the CsPb(Br_x_I_1-x_)_3_ crystallites in the composites exhibited high stability under 0.3 W cm^−2^ illumination. The scale bars in **a** and **d** are 100 nm and 50 nm, respectively. The scale bars in **b** and **e** are 10 nm. Magnified figures of the insets can be found in Supplementary Figure 8

We prepared thin films of mixed-halide perovskites using dual-source thermal evaporation (Methods). Solid precursors PbBr_2_/PbI_2_ and CsBr/CsI were evaporated onto glass substrates followed by thermal annealing for sufficient inter-diffusion and reactions. With a stoichiometry ratio of Pb/Cs in the premixed precursors, polycrystalline thin films of CsPb(Br_x_I_1-x_)_3_ was obtained (Fig. 1a). The perovskite crystal phase was confirmed consistently in the selected area diffraction (SAD) patterns (Fig. 1a, inset), the high-resolution transmission electron microscopy (TEM) images, and the corresponding fast Fourier transformed (FFT) patterns (Fig. 1b). The photoluminescence peak (corresponding to the energy of bandgap) of CsPb(Br_x_I_1-x_)_3_ could be tuned by varying the ratio of Br/I in the premixed precursors (Fig. 1c).

Photoluminescence of all these CsPb(Br_x_I_1-x_)_3_, however, appeared unstable under extended photoexcitation (Fig. 1c). The peak position, regardless of its initial wavelength, redshifted to ~1.87 eV within 10 min of illumination at an intensity of 0.3 W cm^−2^ (compared with an integrated power density of 0.1 W cm^−2^ of the AM1.5 standard solar spectrum). Such redshift, corresponding to a reduction in the bandgap, is consistent with previously reported observations^23^. The initially homogeneous CsPb(Br_x_I_1-x_)_3_ segregated into Br- and I-rich domains with increased and decreased bandgaps, respectively. The energy transfer between neighboring domains makes the small-bandgap species (i.e. the I-rich domains) dominate the photoluminescence after the phase separation^22–24^. The exceptions in our experiments are the samples with very large Br content (Fig. 1c, *X*_Br_ = 0.8 and 0.9), which exhibited photoluminescence from both large- and small-bandgap species (2.4 eV and 1.87 eV, respectively). The amount of the Br-rich domains in this case is likely too large to be quenched completely by the I-rich domains.

The photoinduced phase separation is found to be highly suppressed when the mixed-halide perovskites are created in form of nanocrystals and are embedded in a non-perovskite matrix. We obtained such host-guest structures by simply increasing the relative weight ratio of the Cs-containing precursor (i.e. CsBr/CsI). According to the phase diagram^31^, such variation of the precursor ratio would produce a mixture of CsPb(Br_x_I_1-x_)_3_ and Cs_4_Pb(Br_x_I_1-x_)_6_ when fully reacted. The expected composite structure was confirmed using TEM (Fig. 1d). The electron diffraction pattern was predominately from the hexagonal lattices of the Cs_4_Pb(Br_x_I_1-x_)_6_ single crystal (close to [100]). A faint ring corresponding to the {200} cubic plane of CsPb(Br_x_I_1-x_)_3_ indicated that many tiny crystallites of the perovskite were distributed within the 400-nm electron beam spot (Fig. 1d, inset). The high-resolution TEM image showed that the nanocrystal CsPb(Br_x_I_1-x_)_3_, with an average radius of ~7.5 nm, were embedded in the Cs_4_Pb(Br_x_I_1-x_)_6_ matrix with clear Moiré Fringes (Fig. 1e). The photoluminescence from these isolated, spatially-confined perovskites appeared remarkably stable. After continuous illumination for 5 hours (0.3 W cm^−2^, 365 nm wavelength), the emission wavelength remained unchanged in all the samples regardless of the Br/I mixing ratio (Fig. 1f). Such photo-stability was only reported previously in iodine dominated mixed-halide perovskites (*x* < 0.3) in rare cases^23^.

A thermodynamic model based on nucleation theory^32^ was developed to understand the mechanisms of the high photo-stability observed (Supplementary Note 1, Theoretical Model Based on Nucleation). In this model, whether the mixed-halide perovskites would remain homogeneous or experience phase separation is determined by changes of the Gibbs free energy Δ*G*. The homogenous phase would be stable when Δ*G* is negative.

Δ*G* in the dark (Δ*G*_dark_) is contributed together by the volumetric enthalpy (Δ*h*_mix_), the volumetric entropy term (*T*Δ*s*_mix_) and the cohesive energies Σ*c*_*i*_*W*_*i*_*r*^2^:

1 $$\Delta G_{\mathrm{dark}}(X_{\mathrm{Br}},T) = \frac{4}{3}\pi r^3[\Delta h_{\mathrm{mix}}(X_{\mathrm{Br}},T) - T \cdot \Delta s_{\mathrm{mix}}(X_{\mathrm{Br}},T)] - \mathop {\sum}\nolimits_i{{{c}_{{i}}{W}_{{i}}r^2}}$$

**Fig. 2:**
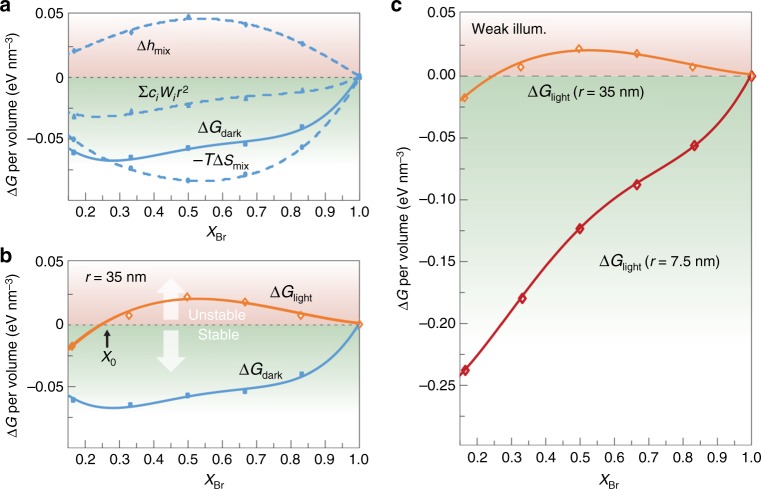
A thermodynamic nucleation model explains the dependence of phase stability on the morphology. **a** The calculated Δ*G*_dark_ per volume (solid line) is negative regardless of the Br content. The dashed lines show the calculated volumetric enthalpy Δ*h*_mix_, volumetric entropy term *T*Δ*s*_mix_, and cohesive energy Σ*c*_*i*_*W*_*i*_*r*^2^. A relatively large grain size was used here (*r* = 35 nm). **b** Under illumination, the calculated free energy ΔG_light_ becomes partially positive assuming the same grain size. A threshold composition *X*_0_~0.3 divides the mixed-halide perovskites into stable (I-rich) and unstable (Br-rich) regions. **c** To mimic the experimental conditions of the CsPb(Br_x_I_1-x_)_3_/Cs_4_Pb(Br_x_I_1-x_)_6_ composites, a small grain size (*r* = 7.5 nm) was assumed and the cohesive energy was considered. The calculated Δ*G*_light_ turns negative, indicating a stable phase of homogenous mixed-halide perovskites

The terms are functions of the average grain size *r*, the temperature *T*, and the relative amount of bromine in the mixed-halide phase *X*_Br_. Based on the quantities calculated using Density Functional Theory (Supplementary Note 2, Computational Methods), Δ*G*_dark_ is found to be negative regardless of the mixed-halide composition (Fig. 2a). A relatively large domain size *r* = 35 nm was assumed in the calculation, but the results would not change qualitatively even if a larger *r* was used. The theoretical predictions are consistent with the experimental results observed in this work and reported previously: the photoluminescence spectra (Fig. 1c), the X-ray diffraction patterns, and the UV-VIS absorption spectra (Supplementary Figure 1) all indicated that the mixed-halide perovskites remained homogeneous in the dark.

Optical illumination could turn the Δ*G* to be favorable for phase separation under certain conditions. The effects of photoexcitation in the model were accounted for by involving the photoinduced polarons^24,33–35^. Polarons could bring in excessive strain energy (Δ*g*_s_) due to the locally shortened and lengthened Pb-halogen bonds (Supplementary Figure 2):

2 $$\Delta G_{\mathrm{light}}(X_{\mathrm{Br}},T) = \Delta G_{\mathrm{dark}}(X_{\mathrm{Br}},T) + \frac{4}{3}\pi r^3 \cdot \Delta g_{\mathrm{s}}(X_{\mathrm{Br}})$$

When Δ*g*_s_ becomes sufficiently large, the strain energy would be released by de-mixing the halogen anions and forming Br- and I-rich domains (i.e. phase separation).

Δ*G*_light_, assuming weak illumination, is found positive when *X*_Br_ > 0.3 (Fig. 2b). The negative Δ*G*_light_ in the I-rich region is indicative of relatively better stability and stemmed from the larger interfacial area with nucleated I-rich domain with a fixed crystal size. The calculated threshold *X*_0_ between the stable and unstable compositions is in reasonable agreement with those observed in our measurements (Fig. 1c) and reported by others previously^23^: photoluminescence from pure CsPb(Br_x_I_1-x_)_3_ redshifted notably under continuous optical illumination when *X*_Br_ > 0.3. The coincidence testified the reasonableness of the physical quantities that we obtained using the nucleation model. It is worth noting that the photoinduced phase separation is reversible in experiments. The shifted photoluminescence would gradually return to its initial wavelength after the optical illumination was removed. The reversibility is also a natural prediction in our theory given that the model was built entirely on the thermodynamic basis.

The phase separation under optical illumination could alternatively be suppressed if the domain size of the mixed-halide perovskites is reduced (Supplementary Note 1.3). The stability could be attributed to the dominating role of the cohesive energy (Σ*c*_*i*_*W*_*i*_*r*^2^) when the surface-to-volume ratio is increased. While all other terms in the model are proportional to the volume of the domains (∝*r*^*3*^), the cohesive energy is a function of the area (∝*r*^*2*^) (Equation (1) & (2) in main text and Supplementary Equation 1 ~ 3). Domains of the new phase (i.e. the iodine-rich perovskite domains) nucleated out of the mixed-halide perovskites would need to reach a certain critical size r* in order for the volume-proportional terms to dominate and the phase separation to prevail (−Δ*G*_light_ < 0). In other words, if the mixed-halide perovskites are confined with an average domain size smaller than r*, nucleation and growth of iodine-rich perovskite domains would never be energy favorable. In that case, the infinitesimal domains of the new I-rich phase would experience dynamic equilibrium between nucleation and dissolution without prevailing phase separation. Experimentally, by confining the nanocrystals of mixed-halide perovskites with *r* = 7.5 nm in the non-perovskite matrix, we were able to limit the size of iodine-rich nucleates. The phase separation then would not occur regardless of the Br content *X*_Br_ (Fig. 2c). The predicted phase stability agrees well with our experiments, in which nanocrystals of the perovskite CsPb(Br_x_I_1-x_)_3_ were spatially confined in the Cs_4_Pb(Br_x_I_1-x_)_6_ matrix with large surface-to-volume ratio (Fig. 1d–f). What also helps to suppress the phase separation is the endotaxial lattice matching between the CsPb(Br_x_I_1-x_)_3_ guest and the Cs_4_Pb(Br_x_I_1-x_)_6_ host matrix^31^. The phase separation becomes further unfavorable because a segregated perovskite domain would increase the energy at the host-guest interfaces and raise the total Gibbs free energy (Supplementary Figure 3b).

### Spectral stability under extremely intensive illumination

**Fig. 3:**
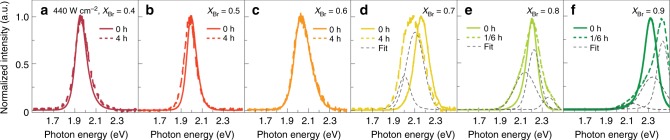
The perovskite composites remained stable under extremely intensive illumination. The solid and dashed lines are spectra obtained from experiments. The thin grey lines show the results of multiple-peak fitting. **a**–**c** Minimal changes were observed in the photoluminescence measured from the samples with *X*_Br_ ≤ 0.6, even after 4 hours of illumination with intensity of 440 W cm^−^^2^. **d**–**f** The sample with higher bromine content (*X*_Br_ ≥ 0.7), which was stable under low-intensity illumination, exhibited redshift and blueshift in the photoluminescence with the strong illumination (440 W cm^−2^)

Remarkably, the tuned bandgap of the CsPb(Br_x_I_1-x_)_3_/Cs_4_Pb(Br_x_I_1-x_)_6_ composites remained stable even under extremely intensive illumination. Using a focused laser beam (λ = 405 nm), we strained the samples optically with an intensity 440 W cm^−2^ (equivalent to 4400 times of integrated intensity of the AM1.5 solar irradiation). No obvious changes were observed in the photoluminescence spectra taken from the samples with *X*_Br_ ≤ 0.6 (Fig. 3a–c). However, with higher bromine concentrations, the samples started to show phase separation under the strong illumination condition (Fig. 3d–f). The correlation between the photo-stability and the composition is consistent with the relative phase stability between the Br- and I-rich perovskites discussed above (Fig. 2c) and in Supplementary Figure 4. Δ*G*_light_ becomes smaller with less Br content (*X*_Br_) and results in a more stable alloy phase. Note that the amount of peak shift appeared noticeably different between the composites and the pure perovskites: the peak in the former settled at higher energy (2.00 eV) compared with the latter (Fig 1c, 1.87 eV). The quantitative difference suggested a smaller degree of phase separation in the composites with a smaller change of iodine concentration in the segregated I-rich domains.

The observed high photo-stability indicates that the cohesive energy is a more dominating factor in stabilizing a homogenous phase. As long as the grain size of the mixed-halide perovskites is sufficiently small, the presumable increase of the cohesive energy associated with the phase separation would dominate over the increase of the photoinduced strain energy, leading to a highly stable homogenous phase even though the high illumination intensity was assumed (Supplementary Note 1.4).

### Temperature dependent photo-stability

**Fig. 4:**
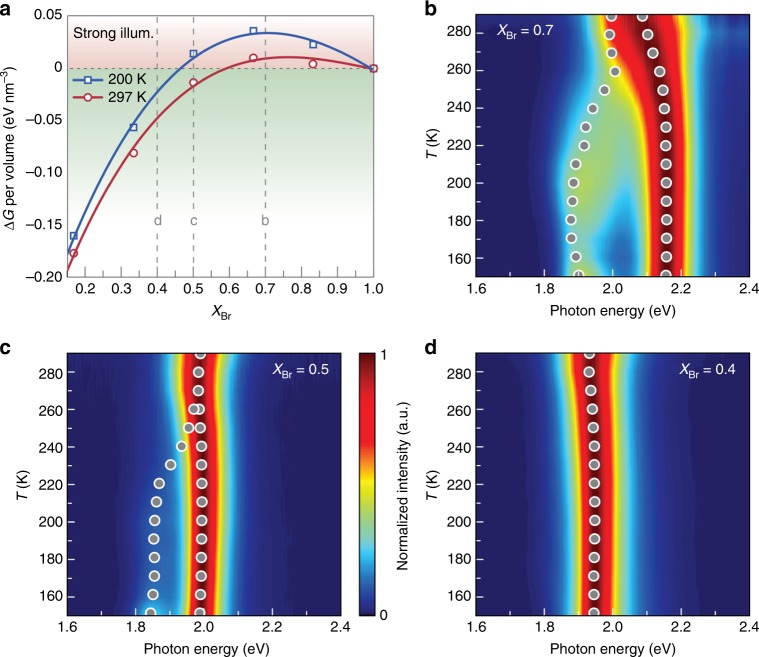
Photo-stability of the perovskites exhibited strong temperature dependence. **a** The calculated Δ*G*_light_ indicates that, under strong illumination, the perovskite in the composites would be less photo-stable as the temperature reduces from 297 K to 200 K. The vertical dash lines correspond to the compositions of samples examined experimentally in **b**–**d**. **b**–**d** Photoluminescence spectra of CsPb(Br_x_I_1-x_)_3_/Cs_4_Pb(Br_x_I_1-x_)_6_ composites with *X*_Br_ = 0.7 (**b**), *X*_Br_ = 0.5 (**c**) and *X*_Br_ = 0.4 (**d**) were measured at temperatures from 150 K to 290 K (increase *T*). Reduced photo-stability, indicated by further split of photoluminescence peaks, was observed at lower temperature in **b** and **c**. The sample with higher iodine concentration in **d** (*X*_Br_ = 0.4) remained consistently stable across the entire temperature range, consistent with other measurements where I-rich perovskites appeared generally more photo-stable. The grey circles in **b**–**d** show the peak positions determined by fitting the measured photoluminescence spectra. **b**–**d** share the same color scale as shown in **c**

Photo-stability of the mixed-halide perovskites, according to the theoretical model with an explicit variable *T* (Equation (1) & (2)), should also be a function of the temperature. In general, Δ*G*_light_ increases when the temperature is reduced (Fig. 4a). Accordingly, an unstable mixed-halide perovskite could experience further phase separation as the temperature is lowered (Fig. 4a, dashed line b). Alternatively, samples that are photo-stable at room temperature could become unstable (Fig. 4a, dashed line c) or remain stable (Fig. 4a, dashed line d) at a lower temperature depending on the level of Br content (*X*_Br_).

We were able to observe such temperature dependence experimentally by measuring the photoluminescence at different temperatures. Samples with three representative compositions *X*_Br_ = 0.7, 0.5, 0.4 (corresponding to the vertical dashed line b/c/d in Fig. 4a) were characterized. Given the high stability of the materials under low-intensity illumination, we had to use focused 405-nm laser light (440 W cm^−2^) in order to reveal the evolution of photoluminescence with the temperature. Upon changing the temperature, no phase separation or abrupt changes have been observed in the initial spectra of photoluminescence, as shown by an example in Supplementary Figure 6. The spectra corresponding to final states were obtained after extended hours of illumination when no further evolution could be observed. For the most Br-rich perovskites (Fig. 4b, *X*_Br_ = 0.7), the photoluminescence peak split at room temperature after continuous optical illumination. The spectral separation between the photoluminescence peaks was found to increase continuously as the temperature was reduced from room temperature to 200 K. The larger spectral split indicated higher iodine and bromine concentrations in the segregated domains, respectively. The reduced photo-stability could also be observed in the perovskite with nearly 1:1 Br/I ratio (Fig. 4c, *X*_Br_ = 0.5). While no spectral shift was observed at room temperature, a new peak corresponding to I-rich domains emerged as the temperature was reduced. Among the three samples, only the one with the highest iodine content remained homogeneous regardless of the temperature (Fig. 4d, *X*_Br_ = 0.4). The agreement between the theoretically predicted and the experimentally measured temperature dependence further validated the model of phase separation based on nucleation. Such phenomena could be attributed to the effect primarily driven by the changes of the entropy, which favor more uniformly distributed bromine and iodine anions (i.e. a homogenous phase) at elevated temperatures, as often observed in alloy systems^32^.

## Discussion

The stabilization of mixed-halide phases did not come at the cost of compromised optical properties. The photoluminescence quantum yields (PLQYs) of the composite thin films remained comparable with the previously reported bright emitters based on mixed-halide perovskites, with the highest reaching 36.8% (Supplementary Figure 7a, *X*_Br_ = 0.5, 620-nm (2.0-eV) emission). The average photoluminescence lifetime was measured as 20~70 nanoseconds (ns), which appeared fairly long among the Cs-based perovskite thin films reported (Supplementary Figure 7b). The addition of non-conductive Cs_4_PbX_6_ may alter the electrical properties from that of the thin films containing pure mixed-halide perovskites. Future studies of electrical properties will be necessary to optimize the optoelectronic functionalities of the inorganic composite perovskites by, for instance, finely tuning the molar ratio between the two component compounds in the composites.

## Methods

### Materials synthesis

An Edwards E306A coating system was used for dual-source evaporation. Cs and Pb salts (CsBr, CsI, PbBr_2_, PbI_2_) were premixed with desirable ratios and used as precursor materials. Glass or thermal oxide substrates were cleaned sequentially by sonication in 1% Alconox precision cleaner solution, acetone, and isopropyl alcohol for 20 min followed by UV ozone treatment for 10 min prior to use. With a base pressure of 6 × 10^–6^ mbar, the two precursor mixtures containing Cs and Pb salts were evaporated alternatingly from separate quartz crucibles. The evaporation rates were controlled 0.1–0.5 Å s^−1^ for the Pb salts and 0.1–0.5 Å s^−1^ for Cs salts. The above steps were repeated until the desired composition and film thickness were obtained (Supplementary Table 1). The evaporated samples were annealed at 150 °C on a hot plate in a nitrogen-filled glove box for 30 min. Cesium bromide (99.9%), cesium iodide (99.9%), lead bromide (98%) and lead iodide (98%) were acquired from Sigma-Aldrich.

### Characterization

A PANalytical X'PERT Pro powder X-ray diffractometer with a Cu Kα source was used to examine the crystal phases of the thin films after thermal annealing. The diffraction patterns were recorded from 2θ = 10° to 45° with a step size of 0.0167° at 0.8° min^−1^. TEM images were acquired using a JEM-ARM200cF operated at 200 kV. Samples for the TEM measurements were prepared by evaporating the thin films directly onto Cu/lacey-carbon TEM grids. Optical absorption spectra were measured using an Agilent Cary 5000 UV-Vis-NIR spectrometer. To avoid the exposure to the moisture in air, the samples were placed in a home-made airtight container with two sapphire windows. Photoluminescence spectra were measured using a HORIBA iHR320 spectrometer, equipped with a HORIBA Synapse back-illuminated CCD. The samples were placed in a vacuum chamber with cryogenic temperature control (Janis ST-500). A continuous-wave diode laser (405-nm) was used as the photoexcitation source. By focusing the laser beam to a 3-µm spot, the optical power density (i.e. intensity) could reach as high as 442.0 W cm^−2^.

### DFT calculation

The Perdew-Burke-Ernzerhof (PBE)^36^ exchange-correlation functional under the Generalized Gradient Approximation (GGA) and the projector augmented-wave (PAW)^37^ formalism were used for the total energy calculation. A plane-wave cutoff energy of 500 eV and a 6 × 6 × 6 k-point mesh was used for all the configurations. The lattice volume and shape, and the atomic positions of each configuration were fully optimized using a quasi-Newton (variable metric) algorithm^38^ to minimize atomic forces below 1.0 meV per Å. The relaxed mixed-halide perovskite configurations at *X*_Br_ = 0.5 were shown in Supplementary Figure 5a, where the degeneracy was five considering symmetry-reduced inequivalent configurations.

To determine the cohesive energy between pure CsPbBr_3_ and CsPbI_3_ *W*_1_ (equivalent to interfacial tension), we constructed a supercell (2 × 2 × 2 of CsPbBr_3_ interfacing with 2 × 2 × 2 of CsPbI_3_) with an interface between the two pure materials, as shown in Supplementary Figure 5b. It is important to note that the cohesive energy contains contributions from: (i) the chemical interfacial energy due to chemical potential difference at the interface, and (ii) the strain energy at the interfaces due to lattice mismatch between the two crystals when they are constrained to the same lateral dimension in the supercell. The same DFT method and parameters mentioned above were used to relax this supercell, as well as to obtain the optimized configuration and the associated total energy at the ground state.

The similar DFT method was used to determine the cohesive energy *W*_2_ between the initial CsPb(Br_x_I_1-x_)_3_ (nanocrystals 113) and Cs_4_Pb(Br_x_I_1-x_)_6_ (matrix 416) phases, as well as the cohesive energy *W*_2_′ between Br-rich CsPbBr_3_ and Cs_4_Pb(Br_x_I_1-x_)_6_ after phase separation. As shown in Supplementary Figure 5c, the interface between the cubic CsPbBr_3_ phase (the (111) surface) and the hexagonal Cs_4_PbI_6_ phase (the (100) surface) is illustrated as an example. The CsPbBr_3_/Cs_4_PbBr_6_, CsPbBr_3_/Cs_4_PbI_6_, CsPbI_3_/Cs_4_PbI_6_ and CsPbI_3_/Cs_4_PbBr_6_ interfaces were all relaxed and equilibrated using DFT calculations based on the reported crystallographic surfaces in an earlier work^31^.

## Supplementary information

Supplementary Information

## Data Availability

The authors declare that all the data supporting the findings of this study are available within the paper (and its supplementary information files). All the other relevant data are available from the corresponding author upon request.
