# Supplementary material for: Suppressed phase separation of mixed-halide perovskites confined in endotaxial matrices
Source: Nat Commun. 2019 Feb 11;10:695. doi: 10.1038/s41467-019-08610-6 (PMC6370784; doi:10.1038/s41467-019-08610-6)
Supplement: Supplementary file 1 — Supplementary Information [file 41467_2019_8610_MOESM1_ESM.pdf]

Supplementary Information for

Suppressed Phase Separation of Mixed-Halide Perovskites Confined in Endotaxial Matrices

Wang et al.

Supplementary Notes:

Supplementary Note 1. Theoretical Model Based on Nucleation

1.1 ΔG_{dark} for System in the Dark (Ground State)

1.2 ΔG_{light} for System under Optical Illumination (Photo-Excited State)

1.3 Suppression of Phase Separation with Dominant Cohesive Energy

1.4 Illumination-Intensity-Dependent ΔG_{light}

Supplementary Note 2. Computational Methods

2.1 Calculation of Volumetric Gibbs Free Energy of Mixing Δg_v for Ground-State $CsPb(Br_xI_{1-x})_3$

2.2 Computation of Cohesive Energy at the Phase-Separated $CsPbBr_3$-$CsPbI_3$ Interface

2.3 Computation of Cohesive Energy at the $CsPbX_3$-Cs_4PbX_6 Interface

Supplementary Note 3. Calculation of Excess Carrier Density in Mixed-Halide Perovskites upon Photoexcitation

Supplementary Note 4. Photoluminescence Quantum yield (PLQY) and Photoluminescence Lifetime (PL lifetime) of Composite Thin Films

Supplementary Note 1. Theoretical Model Based on Nucleation

A thermodynamic model based on nucleation in phase transformation was developed to understand the mechanisms of the high photo-stability observed. In the model, photoexcited polarons, which were reported previously[1,2], tend to trigger the phase separation. The cohesive energy at interfaces, an additional factor that we considered in our model given the unique composite material morphology, is not in favor of separating mixed-halide perovskites into iodine-rich and bromine-rich domains. Particularly, when the interface-to-volume ratio is sufficiently large (i.e. the size of embedded perovskite nanocrystals was sufficiently small), the weight of the cohesive energy can dominate the total energy of the system, which eventually eliminates the photo-induced phase separation.

1.1 ΔG_{dark} for System in the Dark (Ground State)

Based on nucleation theory[3], the change of the free energy ΔG would be contributed together by the volumetric enthalpy (Δh_{mix}), the volumetric entropy (Δs_{mix}) and all the cohesive energies $\Sigma c_i r^2 W_i$. In the dark, $\Delta G = \Delta G_{dark}$ with the initial Br content X_{Br} was expressed as:

$$\begin{aligned}\Delta G_{dark}(X_{Br},T) &= \frac{4}{3}\pi r^3 \Delta g_v(X_{Br},T) - \sum_i c_i W_i r^2 \\ &= \frac{4}{3}\pi r^3[\Delta h_{mix}(X_{Br},T) - T\cdot\Delta s_{mix}(X_{Br},T)] - (1-X_{Br})^{2/3}\cdot 4\pi r^2\cdot W_1 - 4\pi r^2\cdot\Delta W_2(X_{Br})\end{aligned} \quad (1)$$

The coefficient, c_i, of the cohesive energies, W_i, accounts for both $CsPbI_3/CsPbBr_3$ and $CsPb(Br_xI_{1-x})_3/Cs_4Pb(Br_xI_{1-x})_6$ interfaces during phase separation with the values obtained from DFT calculations in Supplementary Note 2 (*Computational Methods*) with W_1 = 0.42 eV nm^{-2} and $\Delta W_2(X_{Br}) = 0.312\cdot(1-X_{Br})$ eV nm^{-2}. In Fig. 2a, for the $CsPb(Br_xI_{1-x})_3$-dominant thin films, only the interface W_1 between $CsPbI_3$ and $CsPbBr_3$ were considered and ΔW_2 (X_{Br}) was treated as zero. The phase diagram of the alloy could be built by calculating the free energy at 297 K as shown in

Fig. 2a in the main text to arrive at similar conclusions of an entropy-driven preference for mixing as in earlier computational studies of mixed-halide perovskites[4-6].

1.2 ΔG_{light} for System under Optical Illumination (Photo-Excited State)

The effects of photoexcitation were accounted for by involving the photoinduced polarons[1,7-9]. The polarons could bring in excessive strain energy (ΔG_s) on top of the ground-state ΔG_{dark} in Supplementary Equation 1 due to the locally shortened and lengthened Pb-halogen bonds (Supplementary Figure 2). When Δg_s (ΔG_s per volume) becomes sufficiently large, the strain energy would be released by de-mixing the halogen anions and forming Br- and I-rich domains (*i.e.*, phase separation). The increased strain energy was calculated as following[3]:

$$\Delta g_s = 4\mu_{mix}(\frac{\delta}{a})^2 - (1-X_{Br})\cdot 4\mu_I(\frac{(a+\delta)-a_I}{a_I})^2 - X_{Br}\cdot 4\mu_{Br}(\frac{(a-\delta)-a_{Br}}{a_{Br}})^2 \qquad (2)$$

where $a = 6.29\text{-}0.46X_{Br}$ / $a_I = 6.29$ / $a_{Br} = 5.83$ Å (lattice constant values from PDF 01-076-8588, 00-054-0752) is the initial lattice constant for the pseudocubic lattice of $CsPb(Br_xI_{1-x})_3$/$CsPbI_3$/$CsPbBr_3$ in dark, $\mu = 6.858\text{-}0.958X_{Br}$ / $\mu_I = 6.858$ / $\mu_{Br} = 5.9$ GPa is the shear modulus of the alloy $CsPb(Br_xI_{1-x})_3$/$CsPbI_3$/$CsPbBr_3$ with the numbers adopted from Cahen et al.[10] and Hantezadeh et al. [11], and δ is the change (shrink or expand) in lattice constant after photoexcitation.

Therefore, $\Delta G = \Delta G_{light}$ in the excited state was written as:

$$\Delta G_{light}(X_{Br},T) = \Delta G_{dark}(X_{Br},T) + \frac{4}{3}\pi r^3 \cdot \Delta g_s(X_{Br}) \qquad (3)$$

The change of lattice distortion to trigger the phase separation in pure $CsPb(Br_xI_{1-x})_3$ (113) thin film with $r = 35$ nm was $\delta = 0.15$ Å (weak illumination condition in the manuscript). This value is in reasonable agreement with previous results calculated based on first principle methods[7]. It is worth noting that the cohesive energy between $CsPb(Br_xI_{1-x})_3$ (113) and $Cs_4Pb(Br_xI_{1-x})_6$ (416) was also considered given the spatial confinement from the $Cs_4Pb(Br_xI_{1-x})_6$ (416) matrix (Supplementary Figure 3).

1.3 Suppression of Phase Separation with Dominant Cohesive Energy

The phase separation under optical illumination could alternatively be suppressed if the domain size of the mixed-halide perovskites is reduced. The stability could be attributed to the dominating role of the cohesive energy $\Sigma c_i r^2 W_i$ when the surface-to-volume ratio is increased. While all other terms in model are proportional to the volume of the domains ($\propto r^3$), the cohesive energy is a function of the area ($\propto r^2$) (Equation 1&2 in main text and Supplementary Equation 1~3). Domains of I-rich phase attempt to nucleate out of the mixed-halide phase (alloy phase). In order for the nucleats to continue to grow (red arrow Supplementary Figure 3c), the domains of I-rich phase would need to exceed a radius r_I^* to get to the critical size r^* with negative ΔG' (positive ΔG_{light} in the manuscript). Otherwise, the free energy reduced due to the nucleation of I-rich domains would not be sufficient to compensate the free energy increased by creation of interfaces (interfaces as shown in Supplementary Figure 3a, 3b); dynamic processes of nucleation and dissolution would then prohibit I-rich phase from growing (yellow arrow in Supplementary Figure 3c). In other words, if mixed-halide phase has a domain size smaller than the r^*, the lowest energy for I-rich phase would be the $r_I = 0$. I-rich phase would never evolve from alloy mixed-halide phase, leading to suppressed phase separation as indicated by the yellow arrow in Supplementary Figure 3c. By confining the nanocrystals with $r = 7.5$ nm in the matrix, experimentally, we are able to limit the size of iodine-rich nucleates into a small value with negative ΔG_{light}. The phase separation would not occur regardless of the Br content X_{Br} under the illumination with low intensity (Fig. 2c).

1.4 Illumination-Intensity-Dependent ΔG_{light}

The average lattice distortion δ is expected to increase under stronger photoexcitation[12-14]. When the intensity of polarons is low, the lattice distortion is limited to the localized area. The perturbed region has a size smaller than the critical size for the iodine-rich domains r_I^* to induce

phase transformation. The average lattice distortion δ in a unit volume is small resulting in a small Δg_s and negative ΔG_{light}. As the intensity increases, the distorted areas start to interact each other. It is then probable for the small nucleates to merge and form larger ones. When the intensity is over the threshold, nucleates with the size exceeding the critical radius of iodine-rich domains r_I^* are formed. δ is increased leading to larger Δg_s and positive ΔG_{light}, inducing phase separation. δ is positively related to the light intensity, so are Δg_s and ΔG_{light} as described in Supplementary Figure 4, before reaching the saturation condition where charge carriers interact strongly with each other. For some Br-rich compositions, the mixed-halide perovskite would remain stable under weak illumination (smaller lattice distortion $\delta = 0.15$ Å), but experience phase separation when the intensity becomes stronger (larger lattice distortion $\delta = 0.20$ Å). With a fixed size of $CsPb(Br_xI_{1-x})_3$ crystal $r = 7.5$ nm ($d = 15$ nm) in $Cs_4Pb(Br_xI_{1-x})_6$ matrix, the asymmetry of ΔG with respect to X_{Br} originated from the smaller cohesive energies with more Br content, as described in Supplementary Equation 1 and 3. It's worth noting that, similar intensity dependent phase separation was reported by Kuno's group[5,15], which is consistent with our model.

Supplementary Note 2. Computational Methods

2.1 Calculation of Volumetric Gibbs Free Energy of Mixing Δg_v for Ground-State $CsPb(Br_xI_{1-x})_3$

We modeled the mixed-halide perovskite $CsPb(Br_xI_{1-x})_3$ at the ground state (in dark) as a statistical ensemble of independent configurations under seven compositions: X_{Br} = 0, 1/6, 1/3, 1/2, 2/3, 5/6, and 1. This was similar to the treatment of a binary alloy system. The mixing enthalpy, $\Delta h_i (X_{Br})$, of each configuration i with ground-state energy, $E_i (X_{Br})$, was defined as:

$$\Delta h_i(X_{Br}) = E_i(X_{Br}) - (1 - X_{Br})E_{CsPbI_3} - X_{Br}E_{CsPbBr_3} \quad (4)$$

where E_{CsPbI3} and $E_{CsPbBr3}$ represented the total energies of the pure compounds at the ground state when $X_{Br} = 0$ and 1, respectively, and both terms were computed in per volume units.

After knowing the mixing enthalpy, Δh_i (X_{Br}), and the degeneracy of each configuration i (due to different combinations of the anion positions under a constant X_{Br}), we could estimate the ensemble (or degeneracy)-averaged mixing enthalpy, Δh_{mix} (X_{Br}, T) as functions of the composition X_{Br} and temperature T accordingly to the Boltzmann distribution:

$$\Delta h_{mix}(X_{Br},T)=\frac{\sum_i \Delta h_i(X_{Br})\exp(-\frac{\Delta h_i(X_{Br})}{k_B T})}{\sum_i \exp(-\frac{\Delta h_i(X_{Br})}{k_B T})} \quad (5)$$

The configuration energies E_i (X_{Br}) were each computed within the framework of Kohn-Sham density functional theory (DFT)[16]. We considered a cubic supercell with 2 × 1 × 1 expansion of a cubic perovskite building block, which corresponds to six halide anions and 10 atoms in total. The total number of configurations for this system was $2^6 = 64$. We studied the cubic polymorph based on our experimentally confirmed fact that they all exhibited the cubic polymorph (Fig. 1a-b in main text, Supplementary Figure 1). For such a cubic inorganic perovskite, the three halide sites are almost equivalent (where the symmetry is greatly enlarged due to the presence of the monoatomic Cs^+ cations), which could reduce the total number of configurations to 21 in total. We took the symmetry-reduced inequivalent configurations and perform a full structural relaxation for each case.

For the DFT total energy calculations, we used the Perdew-Burke-Ernzerhof (PBE)[17] exchange-correlation functional under the Generalized Gradient Approximation (GGA) and the projector augmented-wave (PAW)[18] formalism. A plane-wave cutoff energy of 500 eV and a 6 × 6 × 6 k-point mesh was used for all the configurations. The lattice volume and shape, and the atomic positions of each configuration were fully optimized using a quasi-Newton (variable metric) algorithm[19] to minimize atomic forces below 1.0 meV per Å. The relaxed mixed-halide perovskite configurations at X_{Br} = 0.5 are shown in Supplementary Figure 5a, where the degeneracy was five considering symmetry-reduced inequivalent configurations.

With Δh_{mix} (X_{Br}, T) calculated, the volumetric Gibbs free energy of mixing per unit volume in the dark, Δg_v (X_{Br}, T) could be directly evaluated:

$$\Delta g_v(X_{Br},T) = \Delta h_{mix}(X_{Br},T) - T\Delta s_{mix}(X_{Br},T) \qquad (6)$$

where we estimated Δs_{mix} (X_{Br}, T) assuming the ideal mixing behavior among halide anions:

$$\Delta s_{mix}(X_{Br},T) = k_B[X_{Br} ln X_{Br} + (1-X_{Br}) ln(1-X_{Br})] \quad (7)$$

2.2 Computation of Cohesive Energy at the Phase-Separated $CsPbBr_3$-$CsPbI_3$ Interface

To determine the cohesive energy between pure $CsPbBr_3$ and $CsPbI_3$ W_1 (equivalent to interfacial tension), we constructed a supercell (2 × 2 × 2 of $CsPbBr_3$ interfacing with 2 × 2 × 2 of $CsPbI_3$) with an interface between the two pure materials, as shown in Supplementary Figure 5b. It is important to note that the cohesive energy contains contributions from: (i) the chemical interfacial energy due to chemical potential difference at the interface, and (ii) the strain energy at the interfaces due to slight lattice mismatch between two crystals when they are constrained to have the same lateral dimension in the supercell. The same DFT method and parameters mentioned above were used to relax this supercell, as well as to obtain the optimized configuration and the associated total energy at the ground state, $E_{CsPbI3+CsPbBr3}$. The cohesive energy, W_1, was then calculated as:

$$W_1 = \frac{E_{CsPbI_3+CsPbBr_3} - (E_{CsPbI_3} + E_{CsPbBr_3})}{2A} \qquad (8)$$

where E_{CsPbI3} (2 × 2 × 2) and $E_{CsPbBr3}$ (2 × 2 × 2) represent the DFT-computed total energies of the individual pure compounds at the ground state, each with a cell height that is one half of the supercell used to compute $E_{CsPbI3+ CsPbBr3}$, A represents the relaxed interfacial area, and the factor “2” accounts for the two interfaces due to the periodic boundary condition applied in the DFT calculation. With the assumption that the predicted cohesive energy of the ground-state $CsPbBr_3$/$CsPbI_3$ interface does not change after photoexcitation, we obtained a cohesive energy of

$W_1 = 0.42$ eV nm^{-2} at the $CsPbI_3/CsPbBr_3$ interface with 0.366 eV nm^{-2} contributed from the strain at the interface. The Young's modulus used for strain calculations for $CsPbBr_3$ and $CsPbI_3$ are 15.8 and 14.49 GPa[10,20] and Poisson's Ratios are 0.33 and 0.32 respectively. Compared with the cubic structure, other non-cubic perovskite crystal structures of $CsPbI_3/CsPbBr_3$ such as orthorhombic structure due to different synthesis protocols have lower symmetry. An increase the cohesive energy (chemical + interfacial strain) would be expected in general.

2.3 Computation of Cohesive Energy at the $CsPbX_3$-Cs_4PbX_6 Interface

The similar DFT method based on Supplementary Equation 8 was used to determine the cohesive energy W_2 between the initial $CsPb(Br_xI_{1-x})_3$ (nanocrystal 113) and $Cs_4Pb(Br_xI_{1-x})_6$ (matrix 416) phases, as well as the cohesive energy W_2' between Br-rich $CsPbBr_3$ and $Cs_4Pb(Br_xI_{1-x})_6$ after phase separation. As shown in Supplementary Figure 5c, the interface between the cubic $CsPbBr_3$ phase (the (111) surface) and the hexagonal Cs_4PbI_6 phase (the (100) surface) is illustrated as an example. The $CsPbBr_3/Cs_4PbBr_6$, $CsPbBr_3/Cs_4PbI_6$, $CsPbI_3/Cs_4PbI_6$ and $CsPbI_3/Cs_4PbBr_6$ interfaces were all relaxed and equilibrated using DFT calculations based on the reported crystallographic surfaces in an earlier work[21]. The computed cohesive energies W_2 were 0.373, 0.686, 0.375, and 1.374 eV nm^{-2}, respectively, for the pure $CsPbBr_3/Cs_4PbBr_6$, $CsPbBr_3/Cs_4PbI_6$, $CsPbI_3/Cs_4PbI_6$, and $CsPbI_3/Cs_4PbBr_6$ interfaces. The energy contributions from strain at the interfaces were calculated as 0.179, 0.051, 0.239 and 1.347 eV nm^{-2}, respectively, for the pure $CsPbBr_3/Cs_4PbBr_6$, $CsPbBr_3/Cs_4PbI_6$, $CsPbI_3/Cs_4PbI_6$, and $CsPbI_3/Cs_4PbBr_6$ interfaces. The Young's modulus of Cs_4PbBr_6, Cs_4PbI_6 used for calculations are 14.4 and 11.2 GPa adopted from Hu et al.[22] with Poisson ratios 0.2 and 0.19, respectively.

The $CsPbBr_3/Cs_4PbX_6$ interface is preferred over the interface of $CsPbI_3/Cs_4PbX_6$ due to the much smaller cohesive energy between $CsPbBr_3$ and Cs_4PbX_6. The nucleation of I-rich domains was reported previously in mixed-halide perovskites[1,23]. It is likely that the new I-rich

phase starts to nucleate inside each alloy nanocrystal, making the matrix Cs_4PbX_6 (416) phase interface with a Br-rich $CsPbX_3$ (113) phase in the outer region of the nanoparticle, as illustrated in Supplementary Figure 3. The change of bandgap measured and lattice constant with the composition indicated that 113 and 416 species shared the similar X_{Br} (Fig. 1c&1f, Supplementary Figure 1). Therefore, we could assume that the interface between $CsPb(Br_xI_{1-x})_3$ and $Cs_4Pb(Br_xI_{1-x})_6$ changed into the interface between the Br-rich 113 phase $CsPbBr_3$ and the initial 416 $Cs_4Pb(Br_xI_{1-x})_6$ matrix. Therefore, a cohesive energy change, $\Delta W_2 = (W_2' - W_2)$, is expected upon phase separation in $CsPbX_3$. Since the calculated $W_2(CsPbI_3/Cs_4PbI_6) = 0.375$ eV nm^{-2} is very close to $W_2(CsPbBr_3/Cs_4PbBr_6) = 0.373$ eV nm^{-2}, it is reasonable to assume that $W_2[CsPb(Br_xI_{1-x})_3/Cs_4Pb(Br_xI_{1-x})_6)]$ is a constant of (0.373 + 0.375)/2 = 0.374 eV nm^{-2}, when 113 and 416 are under the same initial X_{Br} value. Therefore, the halide composition dependent change of the cohesive energy was approximately calculated using the linear relation:

$$\begin{aligned}\Delta W_2(X_{Br}) &= W_2^{'}(X_{Br}) - W_2(X_{Br}) \\ &= (1-X_{Br})\cdot[W_2(CsPbBr_3 / Cs_4PbI_6) - W_2(CsPb(Br_{X_{Br}} I_{1-X_{Br}})_3 / Cs_4Pb(Br_{xBr} I_{1-xBr})_6)] \\ &= (1-X_{Br})\cdot(0.686-0.374) eV/nm^2 \\ &= 0.312\cdot(1-X_{Br}) eV/nm^2\end{aligned} \quad (9)$$

The lattices match better between $Cs_4Pb(I_{1-x}Br_x)_6$ and the cubic $CsPb(I_{1-x}Br_x)_3$ compared with other lower-symmetric structures of $CsPb(I_{1-x}Br_x)_3$, such as the orthorhombic structure. Accordingly, the cohesive energy at the $Cs_4Pb(I_{1-x}Br_x)_6$/cubic $CsPb(I_{1-x}Br_x)_3$ interface should be the lowest. Other perovskite structures with lower symmetry (e.g. orthorhombic) would increase the cohesive energy and make the phase separation less favorable in energy. With a large surface-to-volume ratio and the interfaces between $CsPb(I_{1-x}Br_x)_3/Cs_4Pb(I_{1-x}Br_x)_6$ plays a significant role in the phase stability, the cubic $CsPb(I_{1-x}Br_x)_3$ is the preferred structure with the least amount of cohesive energy. Initial PL spectra of $CsPb(Br_{0.5}I_{0.5})_3/Cs_4Pb(Br_{0.5}I_{0.5})_6$ as a function of temperature

showed no signs of phase transition of the cubic-phase perovskite nanocrystals in the composite thin films (Supplementary Figure 6).

Supplementary Note 3. Calculation of Excess Carrier Density in Mixed-Halide Perovskites upon Photoexcitation

Based on our model, it is the density (per volume) of photoexcitation that matters, but not the number of photoexcitation occasions in each domain.

The rate of photoexcitation of electrons and holes in unit volume, regardless of discrete nanocrystals (Cs_4PbX_6-rich) or continuous thin films ($CsPbX_3$-rich), can be expressed the same as:

$$G_n = G_p = \alpha \frac{P_{excitation}}{E_{ph} A} = \alpha \frac{I_{excitation}}{E_{ph}} \quad (10)$$

where α is the absorption coefficient of mixed-halide perovskite; $I_{\mathrm{excitation}}$ is the illumination intensity; E_{ph} is the energy of photons. The excess carrier density is calculated as

$$\delta n = \delta p = \tau G \quad (11)$$

τ is the carrier lifetime, which is estimated as about 20~70 ns from our measurements.

Substitute the numbers with the absorption coefficient[24,25] $\alpha \sim 10^5$ cm^{-1}, $I_{\mathrm{excitation}}$=0.3 W cm^{-2}, E_{ph}=3.39 eV

$$G_n = G_p = 5.53 \cdot 10^{22} cm^{-3} \cdot s^{-1}$$

Substitute τ=20 ns and G from the above calculation, the excess carrier density is calculated as

$$\delta n = \delta p = 1.16 \cdot 10^{15} cm^{-3}$$

Which should be similar to the conditions used in previous reports[1,2,5,26]. Note that the excess carrier density in $CsPbX_3$/Cs_4PbX_6 composites might be under estimated in mixed-halide

perovskite as Cs_4PbX_6 phase also have a small amount of absorption with broad excitation peak centered at E_{ph} = 3.39 eV and pass more charge carriers to $CsPbX_3$ nanocrystals.

For the condition of strong illumination (440 W cm^{-2}), we used a 405-nm laser source. The numbers for the calculation were then changed to

$$G_n = G_p = 8.98 \cdot 10^{25} cm^{-3} \cdot s^{-1}$$

$$\delta n = \delta p = 1.8 \cdot 10^{18} cm^{-3}$$

Supplementary Note 4. Photoluminescence Quantum Yield (PLQY) and Photoluminescence Lifetime (PL lifetime) of Composite Thin Films

The general PLQYs of the all the mixed-halide perovskite thin films are at the level of > 10% with the highest number 36.8% (X_{Br} = 0.5, red color) (Supplementary Figure 7a), comparable with the best PLQY in perovskite thin films. The average lifetime is about 20~70 nanoseconds (ns) (Supplementary Figure 7b), which are comparably long among the Cs-based perovskite thin films. These measured quantities indicate that the stabilization of mixed-halide phases does not come at the cost of compromised optical properties (or photoactivity).

Supplementary References:

1. Bischak, C. G. *et al.* Origin of reversible photoinduced phase separation in hybrid perovskites. *Nano Lett.* **17,** 1028–1033 (2017).
2. Bischak, C. G. *et al.* Tunable Polaron Distortions Control the Extent of Halide Demixing in Lead Halide Perovskites. *J. Phys. Chem. Lett.* **9,** 3998–4005 (2018).
3. Porter, D. A. & Easterling, K. E. *Phase Transformations in Metals and Alloys*. 1–528 (Springer US, Boston, MA, 1992).
4. Brivio, F., Caetano, C. & Walsh, A. Thermodynamic origin of photoinstability in the CH3NH 3Pb(I1–xBrx)3 hybrid halide perovskite Alloy. *J. Phys. Chem. Lett.* **7**, 1083–1087 (2016).
5. Draguta, S. *et al.* Rationalizing the light-induced phase separation of mixed halide organic-inorganic perovskites. *Nat. Commun.* **8,** 200 (2017).
6. Yin, W.-J., Yan, Y. & Wei, S.-H. Anomalous alloy properties in mixed halide perovskites. *J. Phys. Chem. Lett.* **5**, 3625–3631 (2014).
7. Miyata, K. *et al.* Large polarons in lead halide perovskites. *Sci. Adv.* **3,** e1701217 (2017).

8. Bretschneider, S. A. *et al.* Quantifying polaron formation and charge carrier cooling in lead-Iodide perovskites. *Adv. Mater.* **338,** 1707312–8 (2018).
9. Ivanovska, T. *et al.* Long-lived photoinduced polarons in organohalide perovskites. *J. Phys. Chem. Lett.* **8,** 3081–3086 (2017).
10. Rakita, Y., Cohen, S. R., Kedem, N. K., Hodes, G. & Cahen, D. Mechanical properties of APbX3 (A = Cs or CH3NH3; X = I or Br) perovskite single crystals. *MRC* **5,** 623–629 (2015).
11. Afsari, M., Boochani, A. & Hantezadeh, M. Electronic, optical and elastic properties of cubic perovskite CsPbI3: Using first principles study. *Optik* **127,** 11433–11443 (2016).
12. Li, J. *et al.* Dichotomy in ultrafast atomic dynamics as direct evidence of polaron formation in manganites. *Nat. Quant. Mater.* **1,** 221–7 (2016).
13. Wei, T.-C. *et al.* Photostriction of strontium ruthenate. *Nat. Commun.* **8,** 15018 (2017).
14. Wu, X. *et al.* Light-induced picosecond rotational disordering of the inorganic sublattice in hybrid perovskites. *Sci. Adv.* **3,** e1602388–8 (2017).
15. Ruth, A. *et al.* Vacancy-mediated anion photosegregation kinetics in mixed halide hybrid perovskites: coupled kinetic Monte Carlo and optical measurements. *ACS Energy Lett.* **3,** 2321–2328 (2018).
16. Kohn, W. & Shan, L. J. Self-consistent equations including exchange and correlation effects. *Phys. Rev.* **140,** A1133–A1138 (1965).
17. Perdew, J. P., Burke, K. & Ernzerhof, M. Generalized gradient approximation made simple. *Phys. Rev. Lett.* **77,** 3865–3868 (1996).
18. Blochl, P. E. Projector augmented-wave method. *Phys. Rev. B* **50,** 17953–17979 (1994).
19. Pulay, P. Convergence acceleration of iterative sequences - the case of scf iteration. *Chem. Phys. Lett.* **73,** 393–398 (1980).
20. Roknuzzaman, M., Ostrikov, K. K., Wang, H., Du, A. & Tesfamichael, T. Towards lead-free perovskite photovoltaics and optoelectronics by ab-initio simulations. *Sci. Rep.* **7,** 14025 (2017).
21. Quan, L. N. *et al.* Highly emissive green perovskite nanocrystals in a solid state crystalline matrix. *Adv. Mater.* **29,** 1605945–6 (2017).
22. Hu, M., Ge, C., Yu, J. & Feng, J. Mechanical and optical properties of Cs4BX6 (B = Pb, Sn; X = Cl, Br, I) zero-dimension perovskites. *J. Phys. Chem. C* **121,** 27053–27058 (2017).
23. Li, W. *et al.* Phase segregation enhanced ion movement in efficient inorganic CsPbIBr2 Solar Cells. *Adv. Energy Mater.* **7,** 1700946–8 (2017).
24. Maes, J. *et al.* Light Absorption Coefficient of CsPbBr3 Perovskite Nanocrystals. *J. Phys. Chem. Lett.* **9,** 3093–3097 (2018).
25. Fu, Y. *et al.* Selective Stabilization and Photophysical Properties of Metastable Perovskite Polymorphs of CsPbI3 in Thin Films. *Chem. Mater.* **29,** 8385–8394 (2017).
26. Beal, R. E. *et al.* Cesium Lead Halide Perovskites with Improved Stability for Tandem Solar Cells. *J. Phys. Chem. Lett.* **7,** 746–751 (2016).

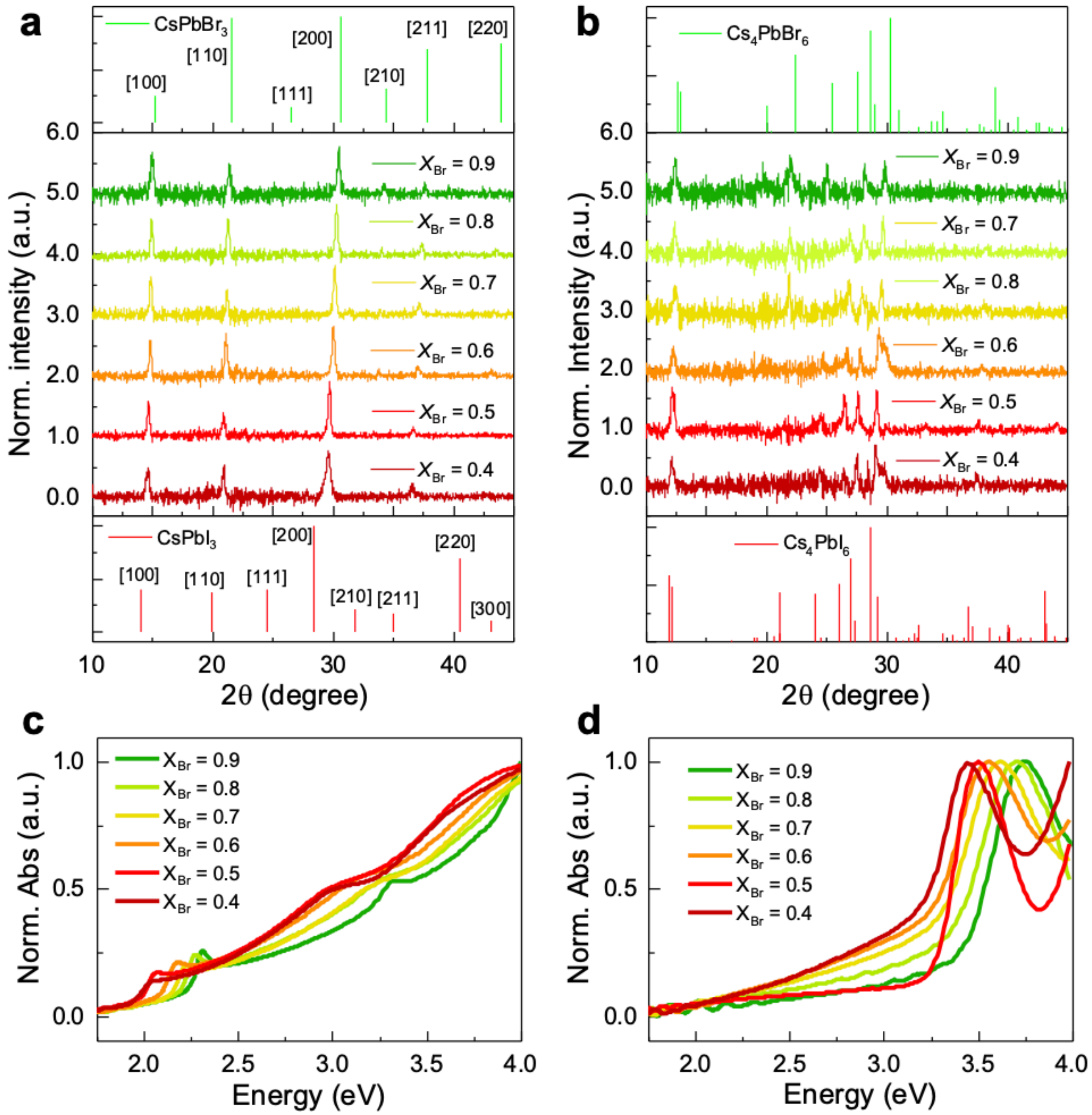

Supplementary Figure 1. XRD and UV-VIS absorption spectroscopy were used to check the composition of the thin films. a, The XRD patterns showed that the lattice constant of cubic-phase perovskites shifted as the ratio of Br/I was varied. **c,** The corresponding spectral edge of the optical absorption (bandgap) changed monotonically to higher energy as the ratio of Br/I was increased. which was consistent with the gradually changed lattice constant in XRD. **b**, The composite thin films were found to be dominated by the host matrix $Cs_4Pb(Br_xI_{1-x})_6$. **d**, Consistently, the optical absorption spectra were also dominated by $Cs_4Pb(Br_xI_{1-x})_6$. The shift of the absorption peak was a result of the change in the ratio of Br/I.

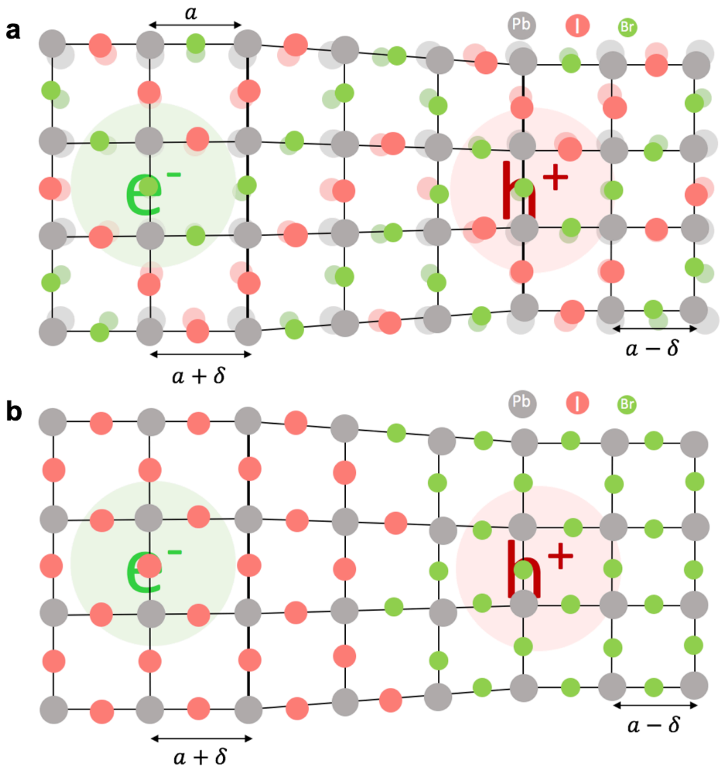

Supplementary Figure 2. The distortion of the lattices in the presence of photo-generated carriers were drawn schematically. Only halogen and Pb ions were displayed for simplicity. The e^- and h^+ represented the positive and negative charge carriers generated by photoexcitation. **a**, In the ground state (dark), the lattices were displayed by the faded-colored dots with a pseudocubic lattice constant a. Under the light illumination, the lattices were distorted due to the interaction between the free carriers and lattices (forming polarons) with the change of constant $a \pm \delta$. **b**, The photoinduced distortion in the lattices would drive the smaller Br^- anions to the lattice-compressed region and the larger I^- anions to the lattice-expanded area to release the localized strain energy. The alloy phase separated into I-rich and Br-rich domains.

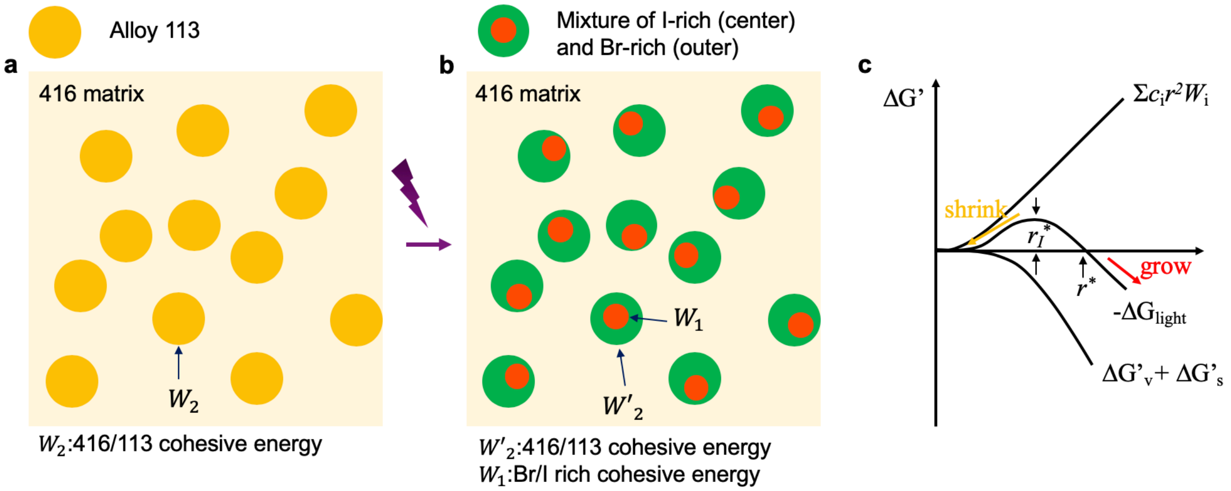

Supplementary Figure 3. All the interfaces of the $CsPb(Br_xI_{1-x})_3/Cs_4Pb(Br_xI_{1-x})_6$ composites before and after photoinduced phase separation were illustrated by schematics. a. Nanocrystals of $CsPb(Br_xI_{1-x})_3$ are embedded in the $Cs_4Pb(Br_xI_{1-x})_6$ matrix with only the $CsPb(Br_xI_{1-x})_3/Cs_4Pb(Br_xI_{1-x})_6$ interface (cohesive energy W_2). **b**, Phase separation occurred under optical illumination with the I-rich domains nucleated in the perovskite nanocrystals. The relative magnitude between the cohesive energy (W'_2 and W_1) is critical to determine the microscopic structures of the phase separation. **c.** The diagram showed the change of free energy as a function of radius r with the competition between the change of volumetric free energy and the cohesive energy.

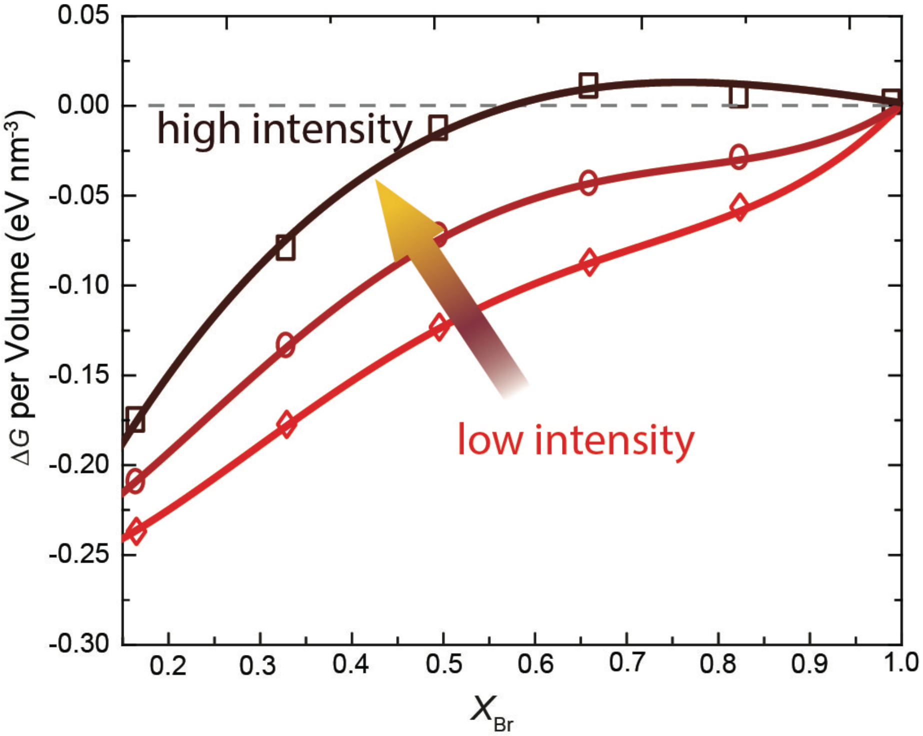

Supplementary Figure 4. ΔG_{light} increased as the illumination became stronger.

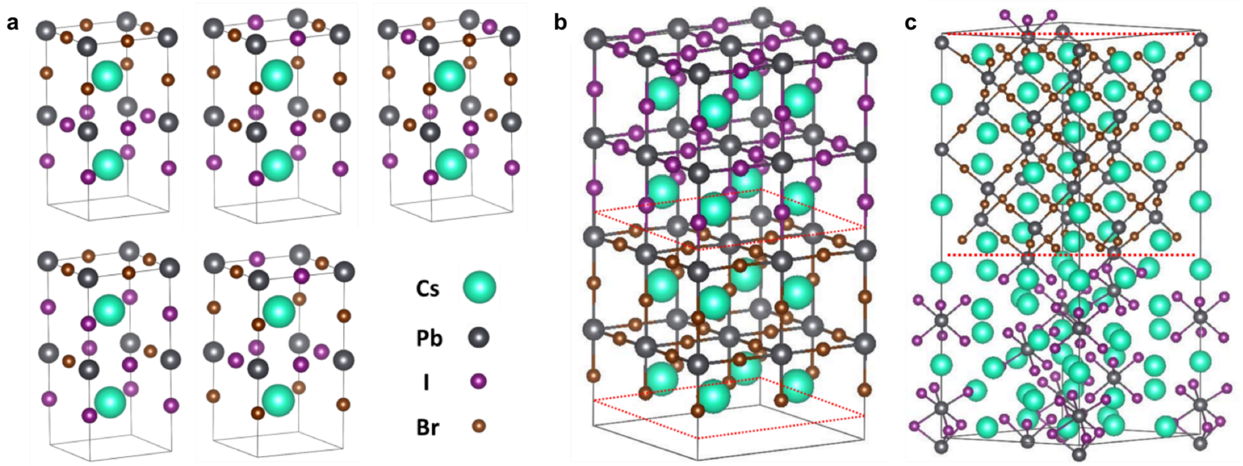

Supplementary Figure 5. DFT-relaxed configurations used for the calculations of various parameters were presented. a, DFT-relaxed symmetry-reduced inequivalent configurations of cubic $CsPb(Br_{0.5}I_{0.5})_3$ with a degeneracy of five were shown. **b**, DFT-relaxed configuration of a supercell showed the $CsPbBr_3$-$CsPbI_3$ interfaces as marked by two dotted red rectangles. **c**, DFT-relaxed configuration of a supercell exhibited the $CsPbBr_3/Cs_4PbI_6$ interface as marked by two dotted red lines. The (111) surface of the 113 phase (top) and the (100) surface of the 416 phase (bottom) form a well-matched interface with two face-sharing PbX_6 octahedra.

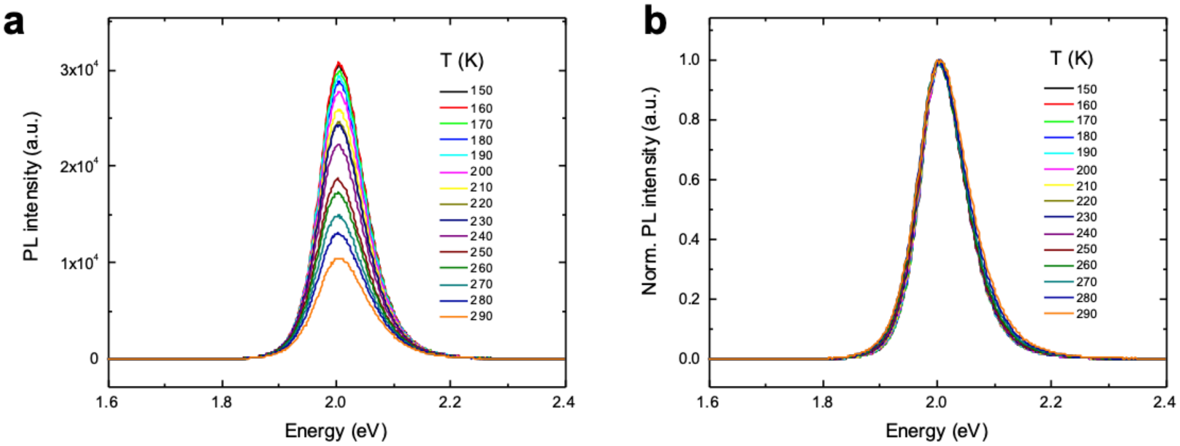

Supplementary Figure 6. Initial PL spectra of $CsPb(Br_{0.5}I_{0.5})_3$/ $Cs_4Pb(Br_{0.5}I_{0.5})_6$ as a function of temperature showed no signs of phase transition of the cubic-phase perovskite nanocrystals in the composite thin film. (a) absolute values of PL. (b) Normalized PL spectra.

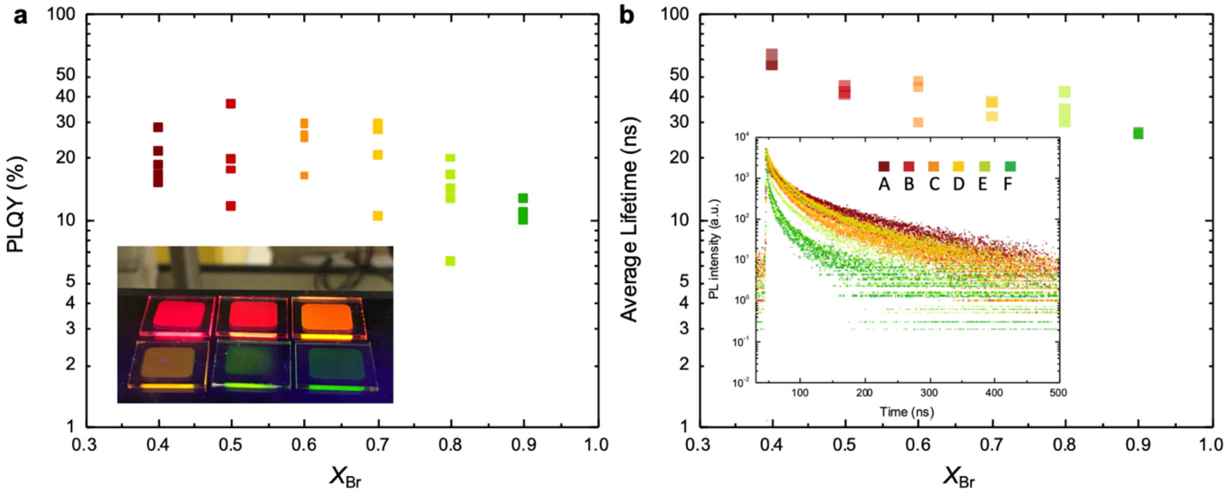

Supplementary Figure 7. The stabilization of mixed-halide phases does not come at the cost of compromised optical properties. a. The general PLQYs of the all the mixed-halide perovskite thin films are at the level of > 10% with the highest number 36.8% (X_{Br} = 0.5, red color). Inset: bright photoluminescence of the $CsPbX_3$/Cs_4PbX_6 composite thin films was observed when excited with a hand-held UV lamp. The photo was taken with the room light on. **b.** The average lifetime is about 20 ~ 70 nanoseconds (ns).

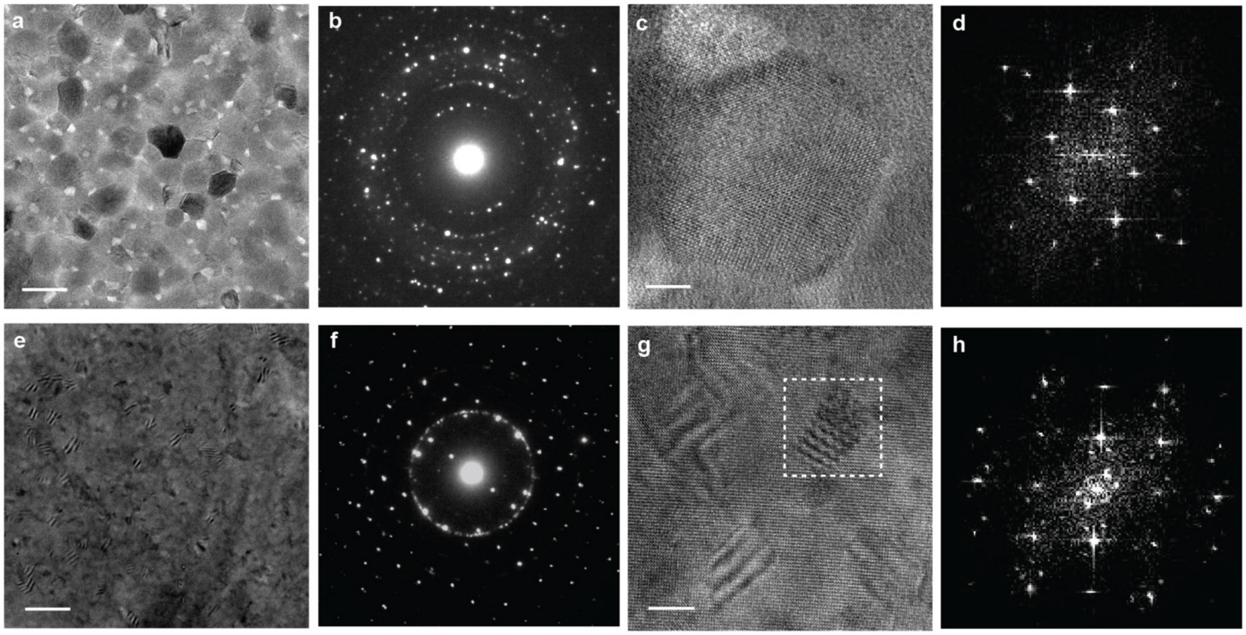

Supplementary Figure 8. Enlarged HRTEM, SAD and FFT patterns of mixed-halide perovskite $CsPb(Br_xI_{1-x})_3$ and $CsPb(Br_xI_{1-x})_3/Cs_4Pb(Br_xI_{1-x})_6$ composite thin films. a. Figure 1a**; b.** enlarged Figure 1a inset; **c.** Figure. 1b**,**; **d.** enlarged Figure 1b inset; **e.** Figure 1d**; f.** enlarged Figure 1d inset; **g.** Figure 1e; **h.** enlarged Figure 1e inset in the main text. The scale bars in (a) and (e) are 100 nm and 50 nm respectively. The scale bars in (c) and (g) are 10 nm.

Supplementary Table 1. Parameters for thermal evaporation

		Cs Source	T (nm)	Pb source	T (nm)	T ratio Cs:Pb
113 rich	X_{Br} = 0.4	CsI	20	$PbBr_2/PbI_2$ 4/1	20	1
	X_{Br} = 0.5	CsI	20	$PbBr_2/PbI_2$ 6/1	20	1
	X_{Br} = 0.6	CsI/CsBr 1/1	20	$PbBr_2/PbI_2$ 6/1	20	1
	X_{Br} = 0.7	CsI/CsBr 3/1	20	$PbBr_2$	20	1
	X_{Br} = 0.8	CsI/CsBr 1/2	20	$PbBr_2$	20	1
	X_{Br} = 0.9	CsI/CsBr 1/3	20	$PbBr_2$	20	1
416 rich	X_{Br} = 0.4	CsI	7.5 x 4	$PbBr_2$	2.5 x 4	3
	X_{Br} = 0.5	CsI/CsBr 5/1	7.5 x 4	$PbBr_2$	2.5 x 4	3
	X_{Br} = 0.6	CsI/CsBr 3/1	7.5 x 4	$PbBr_2$	2.5 x 4	3
	X_{Br} = 0.7	CsI/CsBr 1/1	7.5 x 4	$PbBr_2$	2.5 x 4	3
	X_{Br} = 0.8	CsI/CsBr 1/2	7.5 x 4	$PbBr_2$	2.5 x 4	3
	X_{Br} = 0.9	CsI/CsBr 1/4	7.5 x 4	$PbBr_2$	2.5 x 4	3
